# Targeted next-generation sequencing identified a known *EMD* mutation in a Chinese patient with Emery-Dreifuss muscular dystrophy

**DOI:** 10.1038/s41439-019-0072-8

**Published:** 2019-09-03

**Authors:** Xiafei Dai, Chenqing Zheng, Xuepin Chen, Yibin Tang, Hongmei Zhang, Chao Yan, Huihui Ma, Xiaoping Li

**Affiliations:** 1 School of Medicine, University of Electronic Science and Technology of China, 610072 Chengdu, Sichuan China; 2 Department of Cardiology, Sichuan Academy of Medical Sciences and Sichuan Provincial People’s Hospital, 610072 Chengdu, Sichuan China; 3 Shenzhen RealOmics (Biotech) Co., Ltd., 518081 Shenzhen, China; 4 ZunYi Medical University, 563000 Zunyi, Guizhou China

**Keywords:** Rare variants, Cardiovascular diseases

## Abstract

Emery-Dreifuss muscular dystrophy (EDMD) is a rare X-linked recessive disease characterized by the clinical triad of early childhood joint contractures, progressive weakness in muscles and cardiac involvement and can result in sudden death. Targeted next-generation sequencing was performed for a Chinese patient with EDMD and the previously reported mutation [NM_000117.2: c.251_255del (p.Leu84Profs*7)] in exon 3 of the emerin gene (*EMD*) was identified.

Emery-Dreifuss muscular dystrophy (EDMD) is a rare progressive neuromuscular disease. It is characterized by the clinical trial of (1) early-onset contractures of the elbows, Achilles tendons and posterior neck; (2) slowly progressive muscle wasting and weakness; and (3) cardiomyopathy with cardiac conduction defects, resulting in high-risk sudden death^1–5^. Genetically, EDMD has been associated with *EMD*, *LMNA*, *FHL1*, *SYNE1*, *SYNE2*, *LUMA*, and *SUN1* gene mutations; the most common gene mutations are *EMD*, *LMNA*, and *FHL1*^6,7^. Then, X-linked EDMD (X-EDMD) is caused by mutations in *EMD*, which is located on chromosome Xq28 and is ~2 kb in length and composed of 6 exons^8,9^. Structurally, *EMD*has an N-terminal nucleoplasmic domain consisting of 221 residues, a transmembrane region and a C-terminal tail of 11 residues^10^. X-EDMD, with an incidence of 0.13:100,000^11^, was first described in 1996 by Emery and Dreifuss^12^. Later, autosomal recessive EDMD (AR-EDMD)^13^ and autosomal dominant EDMD (AD-EDMD)^14^ were also reported^15^. Usually, X-EDMD is milder and rarer than AD-EDMD.

A pedigree comprising 5 members from Hubei Province, China, was enrolled in our study. This study was approved by the Sichuan Academy of Medical Sciences and Sichuan Provincial People’s Hospital Trust Ethics Committee. Written informed consent was obtained from all participants. The medical history of the pedigree was queried and recorded in detail. Targeted next-generation sequencing (NGS) was performed.

Qualified genomic DNA of five individuals (the patient, his parents, wife and cousin) was hybridized with the TargetSeq Enrichment Kit (iGeneTech, Beijing, China) to enrich exonic DNA in each library. We performed sequencing of five individuals on the Illumina X-10 (Illumina, Shanghai, China) platform with 150-bp reads independently for each captured library to ensure that each sample had an average coverage of ~300-fold. The probe used in the gene panel can cover all exons of 567 target genes associated with 25 diseases and the 15-bp sequence of exons of their adjacent noncoding regions. The main genes of the panel are shown in the supplemental table.

Samples were aligned to the NCBI human genome reference assembly (hg19) using Burrows-Wheeler Aligner (BWA). Next, we performed Picard MarkDuplicates to mark the duplicate reads to mitigate biases introduced by data generation, such as PCR amplification. The BAM files were processed using the Genome Analysis Toolkit (GATK v3.3) to perform realignment around known indels, and we then recalibrated the base quality scores for the individual base calls in each sequence read.

Germline short variant discovery results from analysis-ready BAM files and produces variant calls. GATK(v3.3) HaplotypeCaller was used to call variants per sample in targeted and flanking regions for each individual to produce a file in GVCF format. We then performed joint genotyping to combine the multi-sample GVCF. Next, we performed GenotypeGVCFs to obtain a multisample genotype for all sites, and finally, a hardfilter was applied to produce the final multi-sample call set with the desired balance of precision and sensitivity.

SnpEff was used to separate SNVs into different functional categories according to their genic location and their expected effect on encoded gene products, based on information from the RefSeq database. All variants were further annotated by the control population of the 1000 Genomes Project (2014 October release), ExAC, EVS, Disease databases of ClinVar, and OMIM. The databases we analyzed are shown in Table 1. If the frequency of the mutation was less than 0.1%, we regarded it as a candidate pathogenic gene. The identified variant was included in ExAC with a frequency of 0.0000114, and it was reasonable to keep the mutation.

**Table 1:** The databases we analyzed

Hgv.c	KG_ALL_AF	KG_EAS_AF	ESP_AF	ExAC_ALL_AF	ExAC_EAS_AF
c.251_255delTACTC	0	0	0	0.0000114315763001	0

The proband was male, age 29, and began having difficulty moving his limb gridle and cervical vertebrae at the age of 5 years. The condition was aggravated when he was 13 years, and normal exercise was limited, with serious elbow contracture and mild scapular winging contracture. The most serious problem was the contracture of the Achilles tendon; he had undergone an operation of lengthening the Achilles tendon without a remarkable effect. At the age of 18, the cardiovascular system was involved and became the remarkable clinical feature. The 12-lead ECG showed junctional escape rhythm accompanied sporadic atrial premature, with an average heart rate of 40 times/min. At the age of 29, the UCG presented progressively serious enlargement of the RA (97.51 × 76.16 mm), RV (51.62 mm), LA (50.50 × 48.13 mm) and LV. However, the patient refused a pacemaker implant.

The results revealed that the proband and his mother shared the same frameshift mutation, [NM_000117.2:c.251_255del (p.Leu84Profs*7)]. The proband was hemizygous (Fig. 1), and his mother was heterozygous (Fig. 1). The mutation was validated by Sanger sequencing. However, his mother presented normal sinus rhythm, and the structure and function of heart were normal by UCG. The proband’s father did not have such a mutation or clinical symptoms (Fig. 1).

**Fig. 1:**
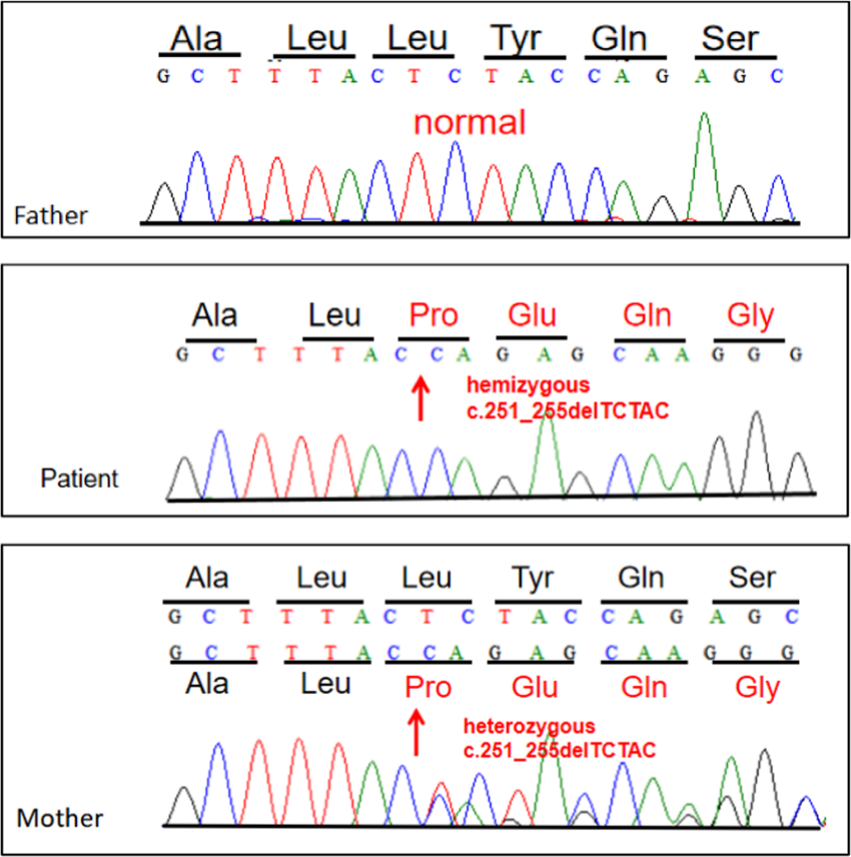
Genomic DNA sequence chromatogram of the proband and his parents. DNA sequence chromatogram of the father as a control. The patient and his mother’s DNA sequence chromatogram indicate NM_000117.2:c.251_255del (p.Leu84Profs*7) in the *EMD* gene. The arrow indicates the position of the TCTAC deletions

In this research, the hemizygous frameshift mutation p.Leu84Profs*7 in the *EMD* gene exon 3 of proband was identified. His mother sharedthe same heterozygous frameshift mutation, and his father did not havethis mutation or clinical symptoms, which indicated that the EDMD in the present study was inherited in the form of X-linked recessive. In 1998, Manilal S et al. reported that the proband and his brother had the same frameshift mutation p.Leu84Profs*7 in *EMD*, but the mutation was not detected in his mother, which was speculated to be a case of germline mosaicism^16^. In contrast to *EMD* mutations reported by Manilal S, we found the same mutation from the patients’mothers. The patient in the present study presented progressively serious contractures of the Achilles tendon and elbow along with increased circulating creatine kinase that began at childhood, and the progressively serious enlargement of the heart and bradycardia.

The *EMD* gene encodes the inner nuclear membrane protein termed emerin^9^. Emerin contains 254 amino acids and is a ubiquitously expressed protein that is located not only in the nuclear envelope but also other tissues and has high mRNA expression in both skeletal muscle and myocardium^17^. Structurally, emerin consists of three parts: a large hydrophilic nucleoplasmic domain (residues 1–222), a transmembrane region (residues 223–243) and a short C-terminal tail (residues 244–254)^18^. Emerin protein is particularly important for the normal function of skeletal muscle and the myocardium. It is anchored to the nuclear envelope proteins on the inner surface of skeletal muscle, myocardium, and smooth muscle membranes, and these interactions imply multiple functions of emerin, including gene expression, nuclear assembly and nuclear envelope stabilization^18^.

In this research, we identified the hemizygous frameshift mutation p.Leu84Profs*7 located in the hydrophilic nucleoplasmic domain of the *EMD* gene, which contains a folded domain and is also observed in other inner nuclear membrane proteins. The consequence of the mutation was a frameshift, resulting in a truncated protein with 84 amino acids, causing a reduction in the size of the hydrophobic nucleoplasmic domain and the complete absence of the transmembrane region and C-terminal tail, which will not only influence the interactions with other proteins such as lamins but also decrease the expression level of emerin. As a result, the emerin protein has difficulty anchoring at the nuclear envelope and cytoskeleton and is subsequently degraded rapidly and losesits biological function of gene expression and stabilizing the nuclear envelope^9^.

Then, in addition to the clinical phenotype in the present research, the proband was misdiagnosed as sick sinus syndrome before genetic testing. A hemizygous frameshift mutation was identified in the proband after NGS. We inferred that the *EMD* mutation may be associated with slowly progressive joint contracture and muscle weakness along with increased circulating creatine kinasebeginningin childhood, along with sinus bradycardia and the progressive enlargement of RA and RV. In this research, the contractures of joint and muscle weakness were in advance of cardiac problems, and the proband presented serious muscle and cardiac involvement, which was different from other patients^18^. Therefore, we speculated that the lack of emerin levels in cardiac tissue may lead to persistent enlargement of RA and RV along with arrhythmia, which was more serious. The loss of emerin in skeletal muscle that may eventually contribute to the contracture of the Achilles tendons and muscle weakness in childhood. In addition, to our surprise, at the age of 18, the proband had more body hair and deeper complexion than his parents, which was speculated to be associated with androgenic augmentation and not reported in other cases. The relationship with the *EMD* mutations remain to be elucidated.

In conclusion, the hemizygous frameshift mutation p.Leu84Profs*7 in *EMD* was identified by NGS, and our study enriches the *EMD* gene mutation database and augments the number of cases of this rare disorder. We provided more information for accurate clinical diagnoses and valuable genetic counseling for families.

## Supplementary information

Supplemental table

## Data Availability

The relevant data from this Data Report are hosted at the Human Genome Variation Database at 10.6084/m9.figshare.hgv.2615.
