# Supplementary material for: Targeted next-generation sequencing identified a known EMD mutation in a Chinese patient with Emery-Dreifuss muscular dystrophy
Source: Hum Genome Var. 2019 Sep 3;6:42. doi: 10.1038/s41439-019-0072-8 (PMC6804839; doi:10.1038/s41439-019-0072-8)
Supplement: Supplementary file 1 — Supplemental table [file 41439_2019_72_MOESM1_ESM.doc]

| category | Disease | Gene number | Detection gene |
| --- | --- | --- | --- |
| cardiomyopathy | Hypertrophic cardiomyopathy | 27 | ABCC9,ACTC1,ACTN2,CALR3,CAV3,CSRP3,JPH2,LDB3,MYBPC3,MYH6,MYH7,MYL2,MYL3,ETCAXN,PLN,PRKAG2,MYLK2,MYO6,MYOZ2,MYPN,NEXN,PLN,PRKAG2,TCAP,TNNC1,TNNI3,TPM1,TTN,VCL |
|  | Dilated cardiomyopathy | 37 | ABCC9,ACTC1,ACTN2,BAG3,CRYAB,CSRP3,DES,DMD,DSG2,DSP,EYA4,FKTN,LAMA4,LDB3,LMNA,MYBPC3,MYH6,MYH7,MYPN,MEXN,PLN,PRDM16,PSEN1,RAF1,RBM2O,SCN5A,SDHA,TMPO,TNNC1,TNNI3,TNNI3K,TTNT2,TPM1,TTN,VCL |
|  | Restrictive cardiomyopathy | 3 | MYPN,TNNI3,TNNT2 |
|  | Arrhythmogenic right ventricular cardiomyopathy | 9 | CTNNA3,DES,DSC2,DSG2,JUP,PKP2,RYR2,TGFB3,TMEM43 |
|  | Noncompaction of ventricular myocardium | 9 | ACTC1.DTNA,LDB3,MIB1,MYBC3,MYH7,PRDM16,TNNT2,TPM1 |
|  | Perinatal cardiomyopathy | 2 | FLT1,PPARGC1A |
| Ion Channel Disease and Arrhythmia | Long QT syndrome | 14 | AKAP9,ALG10,ANK2,CALM1,CALM2,CAV3,KCNE1,KCNE2,,KCNH2,KCNJ5,KCNQ1,SCN4B,SCN5A,SNTA1 |
|  | Short QT syndrome | 3 | KCNH2,KCNJ2,KCNQ1 |
|  | Catecholamine-induced tachycardia | 4 | CALM1,CASQ2,PYR2,TRDN |
|  | Brucellosis | 9 | CACNA1C,CACNB2,GPD1L,HCN4,KCND3,KCNE3,SCN1B,SCN3B,SCN5A |
|  | Familial atrial fibrillation | 13 | ABCC9,GJA5,KCNA5,KCNE2,KCNJ2,KCNQ1,NPPA,NUP155,SCN1B,SCN2B,SCN3B,SCN4B,SCN5A |
|  | Progressive cardiac block | 3 | AKAP10,SCN1B,TNNI3K |
|  | Sick sinus syndrome | 3 | HCN4,MYH6,SCN5A |
|  | Ventricular arrhythmia | 6 | CALM1,CASQ2,GNAI2,RYR2,TECRL,TRDN |
|  | WPW syndrome | 1 | PRKAG2 |
| Other | Numan syndrome | 12 | BRAF,CBL,KRAS,LZTR1,NF1,NRAS,PTPN11,RAF1,PIT1,SHOC2,SOS1,SOS2 |
|  | Marfan syndrome | 1 | PRKAG2 |
|  | Familial aortic aneurysm | 7 | ACTA2,FBN1,MFAP5,MYH11,MYLK,NOTCH1,PRKG1 |
|  | Loeyas-Dietz syndrome | 5 | SMAD3,TGFB2,TGFB3,TGFBR1,TGFBR2 |
|  | Fabry disease | 1 | FBN1 |
|  | Pulmonary hypertension | 5 | BMPR2,CAV1,CPS1,KCNK3,SMAD9 |
|  | Tetralogy of Fallot | 7 | GATA4,GATA6,GDF1,JAG1,NKX2-5,TBX1,ZFPM2 |
|  | atrial septal defect | 8 | ACTC1,CITED2,GATA4,GATA6,NYH6,NKX2-5,TBX20,TLL1 |
|  | Ventricular septal defect | 3 | CITED2,GATA4,NKX2-5 |
|  | Congenital heart disease and multiple congenital abnormal linear skin defects | 3 | COX7B,HCCS,NDUFB11 |

Supplemental table. The main genes and related diseases.
